# Validation of the inventory of Callous-Unemotional Traits among school-going adolescents in Malaysia

**DOI:** 10.1371/journal.pone.0276724

**Published:** 2023-02-16

**Authors:** Aref Ezrin Mohamad Khalil, Hue San Kuay, Maruzairi Husain, Yee Cheng Kueh

**Affiliations:** 1 Department of Psychiatry, School of Medical Sciences, Health Campus, Universiti Sains Malaysia, Kelantan, Malaysia; 2 Department of Psychiatry and Mental Health, Hospital Kemaman, Kemaman, Terengganu, Malaysia; 3 Biostatistics and Research Methodology Unit, School of Medical Sciences, Health Campus, Universiti Sains Malaysia, Kubang Kerian, Kelantan, Malaysia; Boys Town National Research Hospital, UNITED STATES

## Abstract

A key component in the study of antisocial behaviour among adolescents is the presence of callous-unemotional (CU) traits. Among the established tools available to measure CU traits is the Inventory of Callous-Unemotional traits (ICU). To date, there is no validated questionnaire to assess CU traits for the local population. Therefore, there is a need to validate the Malay version of the ICU (M-ICU) so that research can be conducted to explore CU traits among adolescents in Malaysia. The aim of the study is to validate the M-ICU. Two phases of cross-sectional study involving 409 (phase 1 –exploratory factor analysis (EFA), n = 180; phase 2—confirmatory factor analysis (CFA), n = 229) adolescents aged between 13 to 18 years old were conducted from July until October 2020 at six secondary schools in Kuantan district. Participants were selected via multistage random sampling. The ICU was initially translated into Malay language using forward-backward translation procedure by a group of bilingual researchers. Study participants completed the final version of the M-ICU questionnaire and socio-demographic questionnaire. Data was analysed using SPSS version 26 and MPlus software for factor structure validity by performing EFA and CFA. Initial EFA revealed three factors with two items deleted. A further EFA with two factors resulted in the deletion of *unemotional* factor items. Cronbach’s alpha for overall scale improved from 0.70 to 0.74. CFA supported a two-factor solution with 17 items compared to the original English version that has three factors with 24 items. The findings revealed acceptable fit indices (RMSEA = 0.057, CFI = 0.941, TLI = 0.932, WRMR = 0.968). The study revealed that a two-factor model with 17 items of the M-ICU has good psychometric properties. The scale is valid and reliable to measure CU traits among adolescents in Malaysia.

## Introduction

Conduct Disorder (CD) is a highly heterogeneous disorder with a wide variation of severity, course, and aetiology [1]. Its incidence has been associated with criminal involvement and social exclusion as a result and also as an accompanying range of other costs to affected individuals and the society at large. These intangible costs include physical and mental health burden to affected adolescents as adults and the cost to society for providing financial provisions for afflicted individuals [2, 3]. The variability of CD manifestations has led to the difficulties in its diagnosis and management. Previous research has shown strong evidence that the affective component of psychopathy, also called callous-unemotional (CU) traits, defines an important subgroup of children and adolescents with severe conduct problems [4]. In specific, this group of young people differ from other antisocial youth on important biological, emotional, cognitive, and social characteristics [5]. CU traits are also important clinically, being significant enough as to be included in the *Diagnostic and Statistical Manual of Mental Disorders (DSM‐5)* specifier criteria for conduct disorder as ‘with limited prosocial emotions’ [6, 7]. Ever since its inclusion in the diagnostic classification, there has been an increased focus on the best method to assess CU traits [5].

Youths with high scores of CU traits have typical characteristics such as lacking remorse or guilt, having a shallow affect, and a callous disregard for others which predispose them to a tendency to commit crime or violate the law [5, 9]. The increased use of CU traits, separately from other domains of psychopathy, makes it important to ascertain how these traits relate to other personality constructs. These traits are believed to be the precursor to the development of adult psychopathy [8, 9]. In addition, the temperamental features related to CU traits are risk factors for impairments in young people’s conscience development. Extensive empirical research implicates CU traits in the development of severe and persistent antisocial behaviours [10] and having poorer responses to standard interventions for antisocial behaviour [11]. CU traits have emerged from just being a component under the contruct of psychopathy to being examined, itself, as a construct that is worth studying [12]. Thus, studying CU traits could further enhance the current theoretical models that explain the development of severe antisocial behaviour.

Among young people with severe conduct problems, however, it appers that about a quarter (25–30%) of them were found as having heightened level of CU traits [13]. Despite the number, these subgroup of children and adolescents is important to be examined and treated clinically for many significant reasons, which include but not limited to, them showing more severe and more stable conduct problems. In this case, they also show higher level of aggression, their aggression tend to harm the victims more, and their aggression is more goal-oriented–for personal gain or to dominate the other person [14, 15].

Given the importance of assessing youth for their CU traits, especially among those with conduct problems, two widely used scales in research are the Psychopathy Check List- Youth Version (PCL-YV) [16] and the Antisocial Process Screening Device (APSD) [17]. The downside for the PCL-YV, however, is the time needed to complete the measure, which takes around 60–90 minutes by highly trained clinicians. As for the APSD, it is a 20-item scale which can be self-reported by the young person or by their parents or teachers, but only 6 of the items measures CU traits [17], making it hard to confirm the important facets of CU traits. Motivated by this shortcomings from previous measures, Frick developed the Inventory of Callous-Unemotional Traits (ICU) by expanding the CU traits questions on the APSD [18].

One of the most important studies in testing the psychometric properties of the ICU was conducted by Essau et al. on German school-going adolescents (n = 1443) aged from 13 to 18 years old [19]. Exploratory Factor Analysis (EFA) was conducted in order to test for the scale items’ dimensionality. The researchers also conducted a Confirmatory Factor Analysis (CFA) and they found that the data fit a three-factor model best, which are *callousnes*s, *uncaring*, and *unemotional*. This three-factor structure fits both boys and girls well. The Cronbach’s alphas for the factors were .70 for callousness, .73 for uncaring, and .64 for unemotional. The total internal consistency was .77, which was acceptable. In addition, the study also found CU traits to be corelated with conduct problems and psychosocial problems.

Kimonis et al. [20] investigated the psychometric properties of the ICU on 687 undergraduate students. They conducted exploratory principal component analysis (PCA) to examine the factor structure of the ICU and also to examine the internal consistency of this scale. The scree plot was referred to detemine the which factors to rotate–which resulted in a three or four factor solution. Using varimax rotation procedure, two PCAs were done. However, the four-factor solution resulted in five cross-loaded items, indicating the factor loadings were not interpretable. The three factor loading, on the other hand, was interpretable with uncaring factor accounting for 14.3% of the variance, 13.3% for unemotional factor, and 10.0% for callousness. Item 2 and 10 were removed to improve the internal consistency of the total score. The internal consistency of the factors were .77 for uncaring, .80 for unemotional, and .59 for callousness. These factors were also found to be modestly intercorrelated. The study also found ICU score to be correlated with Coldheartedness and Self-Centered Impulsivity factors of the Psychopathic Personality Inventory. In addition, it also correlates with the score of Levenson’s Self-Report Psychopathy Scale.

Kimonis et al. [21] also conducted another validation study for the ICU among 248 young offenders. CFA was conducted using AMOS plugin on SPSS. Again, the results rendered three factors, which were uncaring, callousness, and unemotional. Similar to their other study conducted on undergraduate students, this study also found items 2 and 10 from the callousness factor showed poor item-total correlation and poor loadings. Thus, the two items were removd. The internal consistency for the factors were between .78 to .84 for uncaring, between .71 to .88 for callousness, and between .45 to .60 for unemotional factor. There were moderate correlaton between the factors. The study, additionally found that ICU correlated with aggression, delinquency, and psychophysiological and self-reported indices of emotional reactivity.

A study conducted on a 455 dutch community sample (self-report, parent report, teacher report, and combination of all three) to validate the ICU which was translated into Dutch [22]. CFA was conducted using LISREL framework. In terms of factor structure, the researchers did a comparison between a single-factor model (all items loaded into one factor), a three-factor model (callousness, uncaring, and unemotional), and a three-bifactor model, with all items loaded as one factor, and also on three distinct factors. The three-bifactor model showed the best fit. The Alpha coefficients for the three factors were between the range of “acceptable” to “good”. The study also found that CU traits were inversely correlated with insensitivity to punishment. Agreeableness and Conscientiousness subscales from the Big Five scale were also negatively correlated with ICU scores.

Pechorro et al. [23] conducted a study among 782 Portuguese community youth (*M*_*age*_ = 15.87 years) using both self-reported full version of the ICU and the short version with 12 items. Results from the confirmatory factor analysis showed that the three-factor structure of the full version of the ICU after removing two items were the best fit. For the short version, one item was deleted, resulting to the best fitting two-factor structure (without *unemotional* factor) of the scale. Both versions of the ICU showed adequate internal consistency, convergent validity, discriminant validity, and criterion-related validity.

Among the Chinese samples, Zhang et al. [24] tested the factor structure and psychometric properties of the chinese version of the ICU on 613 chinese detained boys. Results from the confirmatory factor analysis showed that the three-factor model with 24 items from the original ICU scale did not show a good fit to the data. However, the two-factor with *callousness* and *uncarin*g dimensions with only 11-item showed the best fit for the data. Furthermore, the study also compared the two-factor ICU with other instruments for psychopathic traits, including the Antisocial Process Screening Device and the Youth Psychopathic Traits Inventory–which resulted in comparable and significant associations with the relevant criteria of psychopathy, aggression, and empathy.

Another study was conducted by Allen et al. [25] to test the invariance between the British and Chinese samples using the shortened version of ICU (11 item) and the original 24 items ICU. The study included 437 British children aged between 11 to 14 years old from the East of England and 364 Chinese sample between 10 to 13 years old who were attending school in Guandong, China. The internal consistency of the original 24-item ICU and the 11-item ICU in both samples were good for the total and subfactor scores, but not for the *unemotional* factor. According to a recent meta-analysis review, the *unemotional* scale has shown low internal consistency and poor construct validity in past studies [26]. Thus, overall, the 11-item ICU with *callouness* and *uncaring* subscales was the best fit for both British and Chinese samples.

### The present study

Prior validation studies of the ICU scale mostly conducted in Western populations for both adolescents and adults have demonstrated differential factorial structures ranging from two to five factors. The ICU have shown robust reliability within their respective studies with Cronbach’s alpha values for the total ICU scale score ranging from 0.71 to 0.81 [19, 23–26]. Generally, earlier studies tend to favour the three-factor model, in which all the items loaded onto a general factor as well as onto three separately identified factors namely the *uncaring*, *callousness*, and *unemotional* domains, especially in the assessment on adolescents group [18, 27]. These domains are used widely as research tools in studies investigating the development of psychopathic traits and antisocial behaviours [28].

However, some validation studies have stated that the ICU scale with 24 items and fitted into three-factor model generally has a poorer overall fit [14, 22, 29].

A recent meta-analysis conducted by Cardinale and Marsh had reported good internal consistency of the ICU self-report scale with Cronbach’s alpha for the total ICU, α = 0.81; uncaring, α = 0.78; callous, α = 0.73; while the unemotional subscale was slightly lower with α = 0.66 [20]. The meta-analysis also supported strong convergent validity of the ICU with medium to large effect sizes for pooled associations between external measures of psychopathy and the total ICU, r = 0.47, callous subscale, r = 0.43 and uncaring subscale, r = 0.40. However, the association with the unemotional subscale was small with r = 0.16. This pattern of findings was consistent when examining the external validity of the total ICU and its respective subscales with the pooled associations between externalizing outcomes and the total ICU, r = 0.34, callous subscale, r = 0.35, and uncaring subscale, r = 0.29, all had a medium effect size. However, the association between the unemotional subscale and externalizing outcomes was almost zero, r = 0.05. Therefore, this meta-analysis concluded that the total ICU, callous and uncaring subscales has strong internal consistency, convergent validity, and external validity to be utilised for the assessments of CU traits. However, it also voiced concerns regarding the validity and utility of the unemotional subscale to detect non-violent delinquency and aggression.

Similar to Cardinale et al.’s findings, other studies have also consistently illustrated the *unemotional* factor of the ICU to display a relatively weaker psychometric property and low internal consistency as compared to the other two factors which are *callousness* and *uncaring* [18, 22, 30]. This has led to more recent research conducted especially in non-Western samples such as in China and Japan whereby the ICU was revised to a shortened version consisting of only 11–13 items with *callousness* and *uncaring* domains, while the *unemotional* subscale was excluded from the measure [24, 31–34]. This shows the importance of conducting the current study to validate the Malay version for the ICU which can then be used among Malay-speaking population. Thus, the present study hypothesised that the two-factor structure of the ICU with the callousness and uncaring dimensions would be the best fit for the data.

## Materials and methods

### Participants

The study was granted ethical approval from the Human Research Ethics Committee of Universiti Sains Malaysia (USM) with the code USM/JEPeM/19080489. The ethical clearance was valid between 2 January 2020 until 1 January 2021. This cross-sectional study was conducted in randomly selected secondary schools in Kuantan from July 2020 to October 2020. A total of 409 participants (phase 1, n = 180; phase 2, n = 229) aged between 13 and 18 years old who fulfilled all inclusion and exclusion criteria were recruited through a multistage random sampling method. Six schools were selected randomly from a list of schools obtained from the district education office. At each selected school, a total of two to three classes were randomly selected from a list comprising all secondary one to secondary six classes. All students in the selected classes were invited to participate in the study. The inclusion criteria of the present study were any student in government-aided schools under the administration of Kuantan District Education Office; aged between 13 to 17 years old; and able to read and communicate in Malay. The exclusion criteria were any government-aided school that use another language, apart from Malay as the medium of instruction. Special schools for learning, visual, hearing, or other impairments which would disable self-rating for the questionnaire were also excluded from the study.

### Measures

#### Socio-demographic questions

Participants’ socio-demographic characteristics were obtained. We include questions on age, sex, ethnicity, parents’ marital status, household monthly income, parents’ employment status, educational level of parents and family history of mental illness, forensic history, and imprisonment or other chronic illnesses.

#### The Inventory of Callous-Unemotional Traits (ICU)

The original ICU was created by expanding the CU subscale of the Antisocial Process Screening Device (APSD) into a specific measure of CU traits, consisting of 24 items. It was initially developed to tackle concerns on the poor internal reliability of CU subscale scores of other measures of psychopathy adapted for youths, for example, the Hare Psychopathy Checklist: Youth Version [35] and the Antisocial Process Screening Device [17, 36, 37].

The current ICU scale has both self-report and observer-report (rated by parents and teachers) versions that have been used to assess CU traits in a wide range of samples, including both males and females [27, 33]. It has also proved useful in other distinct populations including community, forensic, and clinical samples [18, 27, 38]. The focus of the present study is on the self-rated ICU scale which consists of 24 items encompassing three main domains which are *callousness*, *uncaring*, and *unemotional* factors [30, 39]. All 24 items are measured on a Likert scale ranging from 0 to 3, with 0 showing ‘not at all true’, 1 ‘somewhat true’, 2 ‘very true’, and 3 ‘definitely true.’ A previous meta-analysis conducted by Cardinale and Marsh had reported good internal consistency of the ICU self-report scale with Cronbach’s alpha for the total ICU, α = 0.81; *uncaring*, α = 0.78; *callous*, α = 0.73; while the *unemotional* subscale was slightly lower with α = 0.66 [28]. This meta-analysis supported strong convergent validity of the ICU with medium to large effect sizes for pooled associations between external measures of psychopathy and the total ICU, *r* = 0.47, *callous* subscale, *r* = 0.43 and *uncaring* subscale, *r* = 0.40. However, the association with the *unemotional* subscale was small with *r* = 0.16. This pattern of findings was consistent when examining the external validity of the total ICU and its respective subscales with the pooled associations between externalizing outcomes and the total ICU, *r* = 0.34, *callous* subscale, *r* = 0.35, and *uncaring* subscale, *r* = 0.29, all had a medium effect size. However, the association between the *unemotional* subscale and externalizing outcomes was almost zero, *r* = 0.05. Therefore, as mentioned in the introduction section, this meta-analysis concluded that the total ICU, *callous* and *uncaring* subscales has strong internal consistency, convergent validity, and external validity to be utilised for the assessments of CU traits.

### Procedure

The Youth Self-Report version of the Inventory of Callous-Unemotional Traits (ICU) was translated from the original English version using the following steps [15]:

1. Forward translation from English to Malay version was done by two experts who were native speakers in Malay language. They were invited via email. Both of them carried out the translation independently. These two forward translations were then reconciled into one single consensus translation before being back translated. It was done by the research team. This step was important to resolve discrepancies between the translations. Some words or phrases in the translated questionnaire required alternative translation.
2. Another two bilingual experts who had not seen the original English version did the back translation from the Malay version into the English. The purpose of the backward translation process was to ensure that the quality of the Malay translation is such that the same meaning when the translation is moved back into the English language
3. Two psychiatrists who were competent Malay and English bilingual speakers reviewed both the English translation from Malay and Malay translated version from English before finalising it to Malay version of ICU (M-ICU). It was to detect and deal with any translation discrepancies between the two versions of questionnaire.

The final version of M-ICU was pre-tested among 10 adolescents to ensure clarity and easy understanding for the population it was intended. The result of the pre-test was satisfactory with the final Malay scale produced easily comprehended by the participants, thus no amendment was needed. Due to the copyright of the original version of the ICU, the original version of the inventory can be obtained from the original author (Paul Frick) while the translated version is included in the S1 Appendix.

During the translation process, the main challenge was about to provide language that is equivalent both in its meaning and in the use of idioms between the original version and the translated versions of a questionnaire. Anticipating this problem, we did some arrangement beforehand such as invited two independent translators who were fluent in both languages for forward translation, reconciled the forward translation before back translation by another translator, harmonised the final Malay version of questionnaire, and tested the questionnaire among a small group of adolescents.

After the pre-test, the first phase of data (EFA) was collected, and data was analysed for this phase of study before proceeding with the CFA study. The data collection procedure for both phases were similar, where the researcher visited the school two weeks before the study took place and passed the consent forms to the parents through their children. Two weeks later, written consent forms were collected from the schools. The returned forms were examined and a list of students with parental consent was made. Dates were set for data collection, during schooling hours (i.e., during free period or physical education period). Despite obtaining parental consent, the participants were given the option to choose whether they want to participate in the study. Those who agree were requested to sign an assent form to indicate their willingness to participate in the study. Participants were not given any monetary reimbursement for taking part in this study. Each of them received a pen as a token of appreciation for participation, which was not disclosed until they completed the questionnaire.

### Statistical analysis

The Statistical Package for Social Science (SPSS) version 26.0 software was used to analyse the participants’ sociodemographic data for descriptive statistics, Exploratory Factor Analysis (EFA) and Cronbach’s alpha. The MPlus version 8 software was used to analyse the EFA-derived model by using Confirmatory Factor Analysis. The same analytical method was used by other [29, 40] validation studies as it is strongly recommended for use with ordinal items [41]. EFA and CFA were use to investigate the factor structure validity of the M-ICU.

#### Exploratory Factor Analysis (EFA)

A total of 180 participants enrolled in phase 1 (EFA) study. Principal axis factoring with Promax rotation was performed on the 24 completed items to extract the major contributing factors. Items that had factor loadings less than 0.3 [42] were considered for removal while the number of factors with an eigenvalue more than one was further examined. After deletion of items was done, the factor loading was re-examined and the EFA model was subsequently re-specified. For reliability, the cut off value of > 0.6 was taken for Cronbach’s alpha coefficient to be accepted for each construct’s internal consistency [29].

**Confirmatory Factor Analysis (CFA).** A total of 229 participants were enrolled in the phase 2 (CFA) study. When conducting the model re-specification process, any problematic items with factor loadings less than 0.40 [43] were considered for removal and they were done interactively. The modification index (MI), as suggested by MPlus, was examined, and any items’ residual correlation was added if necessary. All re-specifications of the model were performed after discussion with the team of researchers and adequate theoretical support was established. Following indices were used to assess the fitness of the models: Root Mean Square Error of Approximation (RMSEA) with acceptable level of < 0.08, Weighted Root Mean Square Residual (WRMR) with acceptable level of < 1.0 [44], Tucker–Lewis Fit Index (TLI) with acceptable level of > 0.92 and finally the Comparative Fit Index (CFI) with acceptable level of >0.92 [45]. The construct reliability (CR) of the CFA measurement model for M-ICU scale was calculated by using Raykov’s method [46]. A recommended value for CR is at least 0.70 or higher. Discriminant validity of factors was determined by examining the correlation between the factors from the final CFA measurement model and it is considered acceptable if the value is below 0.85 [47].

## Results

### Socio demographic characteristics of the study variables

Referring to Table 1, for phase 1 (EFA) study, there were 180 participants with a mean age of 14.63 years old (SD = 1.06) and most were males, while the phase 2 (CFA) sample was 229 participants with a mean age of 14.62 years old (SD = 1.05) and more than half were males. Majority of the respondents in both phases were of Malay ethnicity, followed by Chinese, and Indians. Most parents of the respondents in both phases were married and living together. For father’s educational status of the participants, almost half (phase 1) and more than half (phase 2) were educated up to secondary level. While the educational status of the participants’ mother also showed a similar trend. In terms of employment status, almost half of the respondents’ father were employed in the private sector in both study phases. For mother’s employment status, one third of them were either employed as civil servants or were housewives. The mean for the monthly household income was RM 5296.00 for the EFA group while for the CFA group, it was higher at RM 6125.70. Majority of the participants had only one to three siblings while the rest had between four to six siblings. One fourth of the respondents have a family history of chronic illnesses such as diabetes or hypertension.

**Table 1:** Sociodemographic data of respondents for Exploratory Factor Analysis (n = 180) and Confirmatory Factor Analysis (n = 229).

Variables	EFA		CFA	
	Frequency (%)	Mean (SD)	Frequency (%)	Mean (SD)
**Age**		14.63 (1.06)		14.62 (1.05)
13	30 (16.67)		41 (17.90)	
14	55 (30.55)		60 (26.20)	
15	48 (26.67)		74 (32.31)	
16	46 (25.56)		52 (22.71)	
17	1 (0.55)		2 (0.87)	
**Sex**				
Male	94 (52.22)		119 (52)	
Female	86 (47.78)		110 (48)	
**Race**				
Malay	110 (61.11)		145(63.3)	
Chinese	59 (32.78)		75 (32.8)	
Indian	8 (4.44)		7 (3.1)	
Others	3 (1.67)		2 (0.8)	
**Parents’ Marital Status**				
Married and living together	152 (84.44)		199 (86.90)	
Married and separated	7 (3.89)		8 (3.50)	
Divorced	16 (8.89)		20 (8.73)	
Widowed	4 (2.22)		2 (0.87)	
No response	1 (0.56)		0	
	**EFA**		**CFA**	
**Variables**	Frequency (%)	Mean (SD)	Frequency (%)	Mean (SD)
**Father’s Educational Status**				
Primary	9 (5.00)		5 (2.18)	
Secondary	82 (45.56)		118 (51.53)	
Diploma	38 (21.11)		41 (17.90)	
Degree	34(18.89)		38 (16.60)	
Masters/PhD	6 (3.33)		16 (6.99)	
Not relevant	11 (6.11)		11 (4.80)	
**Mother’s Educational Status**				
Primary	6 (3.33)		3 (1.31)	
Secondary	96 (53.33)		112 (48.91)	
Diploma	35 (19.44)		55 (24.02)	
Degree	30 (16.70)		44 (19.21)	
Masters/PhD	9 (5.00)		12 (5.24)	
Not relevant	4 (2.22)		3 (1.31)	
**Father’s Employment Status**				
Government staff	29 (16.11)		44 (19.21)	
Private staff	82 (45.56)		101 (44.10)	
Businessman	6 (3.33)		18 (7.86)	
Retired	9 (5.00)		10 (4.37)	
Self-employed	34 (18.89)		31 (13.54)	
Not relevant	20 (11.11)		25 (10.92)	
**Mother’s Employment Status**				
Government staff	57 (31.67)		77 (33.63)	
Private staff	38 (21.11)		49 (21.40)	
Housewife	67 (37.22)		76 (33.19)	
Businesswoman	4 (2.22)		1 (0.43)	
Self-employed	4 (2.22)		8 (3.49)	
Unemployed	1 (0.56)		0	
Retired	1 (0.56)		3 (1.31)	
Not relevant	8 (4.44)		15 (6.55)	
**Monthly Household Income** [48]		5296.0 (5254.3)		6125.7 (6577.0)
<4,850 4,850–10,959 > 10,959	107 (59.44) 50 (27.78) 13 (7.22)		124 (54.15) 61 (26.64) 29 (12.66)	
Missing	10 (5.56)		15 (6.55)	
**Total Number of Siblings**		3.52 (1.782)		3.48 (1.703)
1–3 4–6 7–9 ≥ 10	97 (53.90) 69 (38.33) 8 (4.44) 2 (1.11)		121 (52.84) 91 (39.74) 11 (4.80) 0	
Missing	4 (2.22)		6 (2.62)	
**Family History of**				
Psychiatric disorder	2 (1.11)		3 (1.31)	
Criminal history	4 (2.22)		5 (2.18)	
Imprisonment	7 (3.90)		5 (2.18)	
Other chronic illness	42 (23.33)		52 (22.71)	
No relevant family history	125 (69.44)		164 (71.62)	

### Exploratory Factor Analysis (EFA) results of the M-ICU

The initial principal axis factoring analysis of all 24 items in ICU indicated sampling adequacy thus a reliable estimate from our current model. The computed Kaiser–Meyer–Olkin (KMO) value of 0.73 was considered good and the Bartlett’s test of sphericity was significant (p < 0.001), thus supporting the validity of our EFA model. The items were analysed using EFA to explore the number of factors and were found to have eight domains with total variances of 61.56%. We investigated the eight domains and their loaded items and found that they did not fit into the theoretical construct of the ICU. Therefore, we proceeded by re-fitting the number of factors to two and three respectively, as this construct was most in tandem with the theoretical basis of ICU. Subsequently, the three factors extracted were named *uncaring* (factor 1*)*, *callousness* (factor 2), and *unemotional* (factor 3) and appeared to have eigenvalues above 1 and cumulatively explained 37.09% of all responses. All items were arranged based on the factor loading under the three factors that were extracted in this study. Two items were deleted: Q10 due to negative loading (not due to reverse scoring issues) and item 6 due to cross-loading into two factors. The Cronbach’s alpha for the overall scale of ICU-22 was 0.70 with 0.79 for *uncaring*, 0.67 for *callousness*, and 0.61 for *unemotional* factor respectively, which indicates acceptable reliability.

The scree plot was used to determine the number of factors to rotate (plot suggested two factor solution to be retained), in addition to the poor reliability, and negative correlation noted between the *unemotional* factor and the other factors in the M-ICU, we repeated the EFA with two factor models and it showed that both factors resulted with eigenvalues more than 1 while the cumulative variance was 28.93%. The two-factor scale resulted in the deletion of five items in total, items 6 and 10 due to negative loading, and items 1, 14 and 19 (which were initially in the *unemotional* scale of the three-factor ICU) were dropped due to poor factor loading into the remaining two domains. The internal consistency (Cronbach’s alpha) of the general scale also showed improvement with an α value of 0.74 after the removal of the *unemotional* factor. Thus, all these items were subsequently deleted. The researchers decided to maintain item 20 ‘*I do not like to put the time into doing things well*’ in the EFA models for both 2-factor and 3-factor despite its factor loading reducing to below 0.30 after deletion of other problematic items such as items 6 and 10. The factor loadings for the two-factor EFA are presented in Table 2 whereby the two remaining factors were *uncaring* and *callousness*. The Cronbach’s alpha for the overall scale of two-factor ICU improved to 0.74 while for *uncaring* and *callousness* factor were 0.79 and 0.67 respectively, which indicated acceptable reliability. The three-factor and two-factor EFA models were then further tested using CFA.

**Table 2:** Factor loading, Cronbach’s alpha value for 3-factor EFA and 2-factor EFA final models.

Constructs/items	3-factor	2-factor
	Factor loading	Factor loading
Uncaring:		
Q3—I care about how well I do at school or work.**	0.64	0.62
Q5—I feel bad or guilty when I do something wrong.**	0.46	0.46
Q8—I am concerned about the feelings of others.**	0.60	0.61
Q13—I easily admit to being wrong.**	0.49	0.49
Q15—I always try my best.**	0.69	0.69
Q16—I apologize (“say I am sorry”) to persons I hurt.**	0.57	0.56
Q17—I try not to hurt others’ feelings.**	0.53	0.53
Q23—I work hard on everything I do.**	0.58	0.58
Q24—I do things to make others feel good.**	0.48	0.48
Cronbach’s alpha	0.79	0.79
Callousness:		
Q2—What I think is “right” and “wrong” is different from what other people think.	0.32	0.33
Q4—I do not care who I hurt to get what I want.	0.42	0.44
Q7—I do not care about being on time.	0.53	0.54
Q9—I do not care if I get into trouble.	0.37	0.38
Q11—I do not care about doing things well.	0.39	0.41
Q12—I seem very cold and uncaring to others.	0.57	0.56
Q18 –I do not feel remorseful when I do something wrong.	0.59	0.60
Q20—I do not like to put the time into doing things well.*	0.24	0.28
Q21 –The feelings of others are unimportant to me.	0.51	0.51
Q22 –I hide my feelings from others.	0.37	0.31
Cronbach’s alpha	0.67	0.67
Unemotional:		
Q1 –I express my feelings openly.**	0.65	-
Q14 –It is easy for others to tell how I am feeling.**	0.44	-
Q19 –I am very expressive and emotional.**	0.63	-
Cronbach’s alpha	0.61	-
Overall Cronbach’s alpha	0.70	0.74

Note

*factor loading reduced to below 0.30 after removal of problematic items from the initial EFA model (24 items), however researcher decided to keep the item for further analysis in CFA.

**Reverse scored items.

### Validity by Confirmatory Factor Analysis (CFA)

CFA was used to examine the validity of the M-ICU. CFA was initially done on the 22-item three-factor EFA model revealed that fit indices for Model-1 were not within acceptable threshold values except for the root mean square error of approximation (RMSEA). To improve the fit indices, factor loadings of each item were then inspected. Several model re-specifications were done interactively by deleting two problematic items from the *callousness* factor (Q2 and Q22) due to very low factor loadings of 0.01 and 0.31 respectively, resulting in Model-2. The fit indices were improved marginally but fit indices of CFI, TLI and WRMR still did not reach the acceptable threshold value. The MI was examined, and the value indicated that by adding correlation between items’ residual (Q23 and Q15), the model fitness would improve. After discussion among the researchers, the Model-2 was re-specified and resulted in Model-3. Model-3 fit the data well with all fit indices achieving the acceptable threshold value (see Table 3). The two-factor model (without *unemotional* factor) as reported in EFA results was tested in Model-4. The fit indices were not within the acceptable threshold value for CFI, TLI and WRMR. Two problematic items, Q2 (0.31) and Q22 (<0.01) with low factor loading were identified and were removed from the model iteratively. The fit indices were improved but still CFI, TLI and WRMR were not within the acceptable threshold value. Model-5 was re-specified by using item residual correlation (Q23 and Q15) and this resulted in Model-6. This is in line with Marais [49] idea in his chapter which indicates that “Residual correlations that are high indicate a violation of the local independence assumption, and this suggests that the pair of items have something more in common than the rest of the item set have in common with each other”. Model-6 fit the data well with all the fit indices achieving the recommended threshold value (see Table 3). All items achieved satisfactory factor loading to their respective factors. Almost all items achieved a factor loading of more than 0.5; except for item 5 whose value (for two-factor and three-factor models) was slightly lower than 0.5.

**Table 3:** Summary for M-ICU model fit indices (n = 229).

CFA model	RMSEA (90% CI)	CFI	TLI	WRMR
3-factor model:				
Model-1^a^	0.065 (0.056, 0.075)	0.877	0.863	1.201
Model-2 ^b^	0.058 (0.047, 0.069)	0.919	0.908	1.058
Model-3^c^	0.051 (0.039, 0.062)	0.939	0.930	0.977
2-factor model:				
Model-4^d^	0.075 (0.064, 0.085)	0.876	0.860	1.232
Model-5^e^	0.067 (0.054, 0.079)	0.919	0.907	1.068
Model-6^f^	0.057 (0.044, 0.070)	0.941	0.932	0.968

^a^ without items Q6 and Q10 based on EFA 3-factor model

^b^ without items Q6, Q10, Q22, Q2

^c^ without items Q6, Q10, Q22, Q2 and added correlated residuals on items Q23 and Q15

^d^ without items Q1, Q6, Q10, Q14, Q19 based on EFA 2-factor model

^e^ without items Q1, Q2, Q6, Q10, Q14, Q19, Q22

^f^ without items Q1, Q2, Q6, Q10, Q14, Q19, Q22, added correlated residuals on items Q23 and Q15

Table 4 shows the standardised factor loadings for Model-3 and Model-6 that exceeded the threshold value of 0.40. The CR values for all the factors in Model-3 and Model-6 were greater than 0.70, which indicated acceptable construct reliability.

**Table 4:** Standardised factor loadings and construct reliability (CR) for Model-3 and Model-6.

Constructs/items	Model-3, 3-factor		Model-6, 2-factor	
	λ	CR	λ	CR
Uncaring:		0.83		0.83
Q3—I care about how well I do at school or work.*	0.63		0.64	
Q5—I feel bad or guilty when I do something wrong.*	0.46		0.48	
Q8—I am concerned about the feelings of others.*	0.72		0.72	
Q13—I easily admit to being wrong.*	0.51		0.5	
Q15—I always try my best.*	0.73		0.73	
Q16—I apologize (“say I am sorry”) to persons I hurt.*	0.66		0.65	
Q17—I try not to hurt others’ feelings.*	0.69		0.69	
Q23—I work hard on everything I do.*	0.56		0.57	
Q24—I do things to make others feel good.*	0.72		0.72	
Callousness:		0.84		0.84
Q4—I do not care who I hurt to get what I want.	0.77		0.77	
Q7—I do not care about being on time.	0.59		0.59	
Q9—I do not care if I get into trouble.	0.56		0.57	
Q11—I do not care about doing things well.	0.50		0.5	
Q12—I seem very cold and uncaring to others.	0.58		0.59	
Q18 –I do not feel remorseful when I do something wrong.	0.84		0.83	
Q20—I do not like to put the time into doing things well.	0.58		0.56	
Q21 –The feelings of others are unimportant to me.	0.57		0.56	
Unemotional:		0.73	-	-
Q1 –I express my feelings openly.*	0.57		-	-
Q14 –It is easy for others to tell how I am feeling.*	0.65		-	-
Q19 –I am very expressive and emotional.*	0.84		-	-

Note: λ = standardised factor loading, CR = construct reliability, all factor loadings were statistically significant at p < .050. Cronbach α for final model 6 with 17 items was 0.81.

*Reverse scored items.

### Discriminant validity

**Fig 1:**
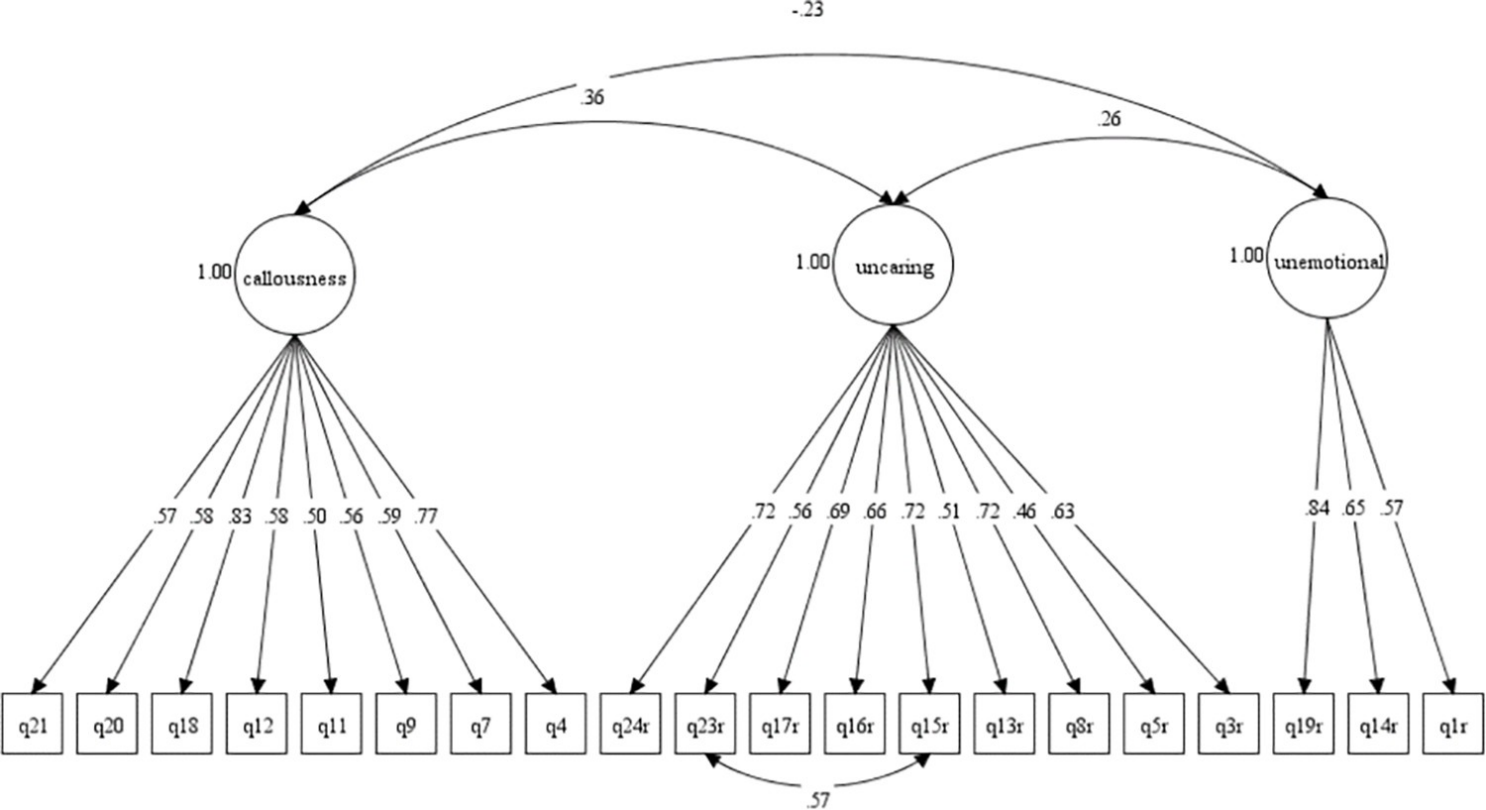


**Fig 2:**
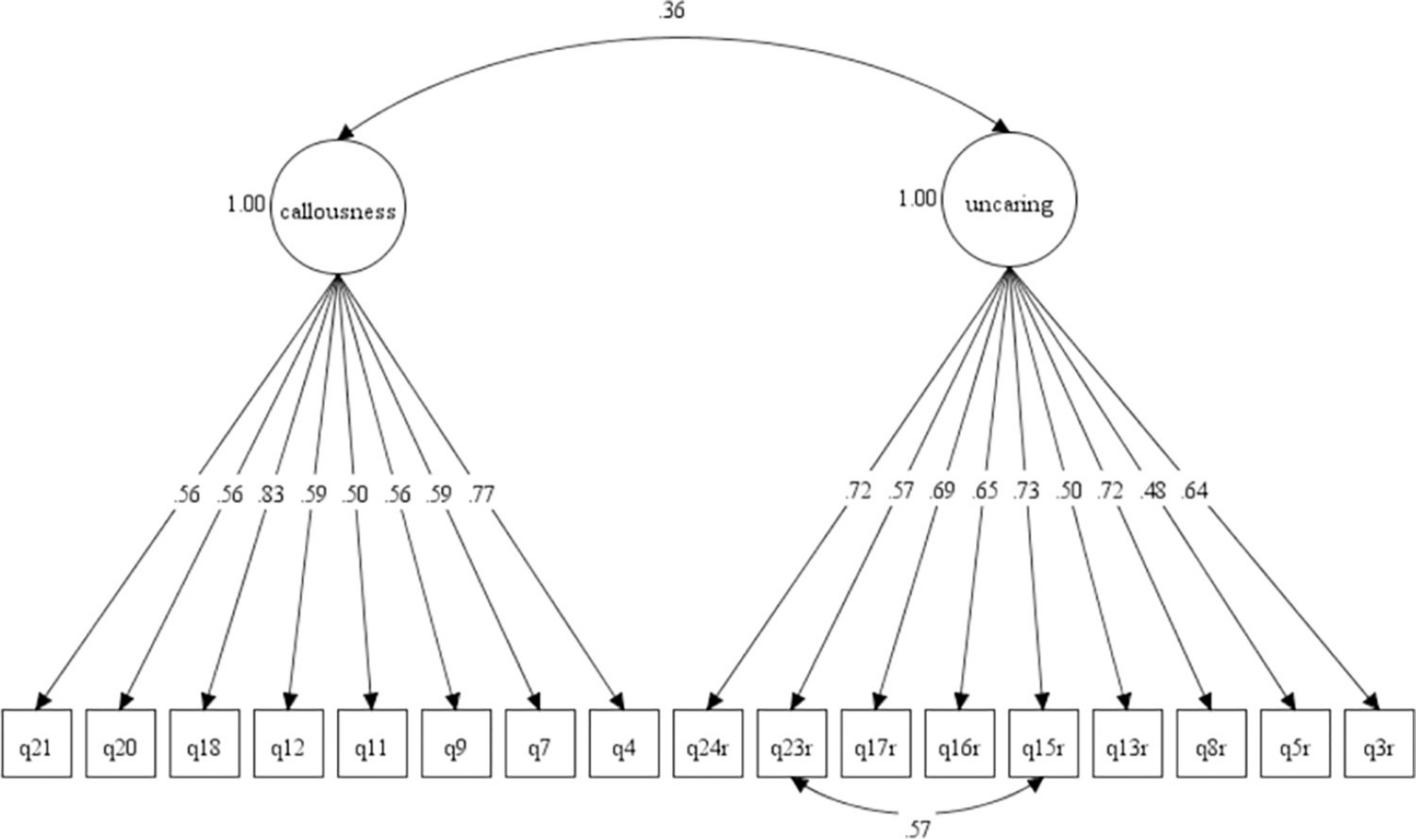


Figs 1 and 2 show the Model-3 and Model-6 in CFA diagram. The standardised correlation between the factors in Model-3 and Model-6 were low with values below 0.85. The results supported good discriminant validity for domains in the M-ICU scale. Based on the CFA results, we compared the final model of both 3-factor (Model-3) and 2-factor (Model-6) models. We found that the two-factor model produced better goodness of fit indices than the three-factor model of M-ICU.

## Discussion

The concept and investigation of CU traits among adolescents in Malaysia is still relatively new. To the best of the authors’ knowledge, this is the first study with the aim to extensively examine the factor structure of the ICU among the adolescent population in Malaysia. Thus, validating the M-ICU would be an essential steppingstone in further discovering the prevalence of CU traits and other associated factors for aggression and delinquent behaviour among young people in Malaysia.

In comparison to the original ICU scale with the most popular three-factor construct, the initial EFA of the current study also produced a three-factor construct with the removal of two items, namely Q6 *‘I do not show my emotions to others’* and Q10 *‘I do not let my feelings control me*.*’* These two items described the emotional regulation of adolescents which could still be in the developmental stage, even more so for children with potential conduct issues who are the targeted group for this ICU scale. This has also been shown in other prior studies which stated item 6 has regularly demonstrated poor factor loading and has consistently been removed from the final versions of other validated ICU scales [33, 50].

The remaining items loaded onto three distinct factors with nine, ten and only three items loading into the *uncaring*, *callousness*, and *unemotional* subscale, respectively. Reliability estimates for the M-ICU are consistent with findings from previous studies which indicate that the internal consistency of the measure is generally acceptable. Although past studies, for example, Essau et al. [19] and Roose et al. [22] have found five items to load into the *unemotional* subscale (items 1, 6, 14, 19, and 22), the present study found that the internal consistency for the *unemotional* factor which consists of only three items was markedly lower than the other two factors. This finding was supported by multiple previous studies which all showed poor internal consistency of the *unemotional* factor [9, 22, 41]. The *unemotional* factor displayed unexpected and negative associations with other theoretically related variables such as with aggression measures, which was evident in past studies. For instance, a recent meta-analysis by Cardinale and Marsh [26] also showed that the pooled association between the *unemotional* subscale and with total CU scores assessed with other measures was small with r = 0.16. Meanwhile, the association between total ICU scores, *uncaring* and *callousness* subscales had medium to large effect sizes for association with psychopathy scores with r value ranging from 0.40 to 0.47.

Houghton and colleagues [50] argued that for children and adolescents, they may have difficulties to appreciate the (affective) emotions of others, and that “they may not have enough experience to be able to attribute these emotions to themselves or others” [42]. From that study, coupled with the findings of the present study, it could be possible that self-reported *unemotional* items may not contribute to differentiating between children with high or low CU traits. In fact, according to Kimonis et al., the *unemotional* factor may be tapping into a distinct construct from CU traits and thus needs to be examined in further detail in future studies to determine the relevance to include them as part of future (self-report) versions of the ICU.

In the EFA, the deleted items that would constitute the *unemotional* construct which are item 1 *‘I express my feelings openly’*, item 14 *‘It is easy for others to tell how I am feeling’*, and item 19 *‘I am very expressive and emotional’* all reflect the element of concealing emotions (once they are reverse scored). This could indicate that Malaysia’s adolescent population tend to be more reserved in their emotional expression, thus leading them to think they are indeed expressing appropriate level of emotion. In other words, they may not see themselves as being *unemotional* although they could be scoring higher on the *callous* and *uncaring* factors. A closer look at the wording in these *unemotional* items would also reveal that most of the questions imply the outward expression of emotions rather than experiencing them, which could contribute to this factor having low internal consistency [28].

To date, there have been more recent studies that have eliminated the *unemotional* factor entirely and produced a shortened version of the ICU (SF-ICU) with the number of items ranging from 10 to 13 items with most of these studies maintaining a bi-factor model. It is noteworthy to mention that the majority of these newer studies were conducted in mostly Asian populations such as China and Japan [24, 25, 31, 33, 34, 51]. This may also indicate that young people from these Asian countries (i.e., China, Japan, and Malaysia) tend to be more reserved when dealing with their emotions which could reflect the more conservative and reserved Asian cultures that are shared between these nations.

Regarding the retaining of item 20, this was done after discussion between the researchers and noting from prior validation studies that item 20 is not commonly seen to be associated with both item 6 and 10 that were deleted initially or to load together with items 1, 14 and 19 from the *unemotional* construct [52]. It is interesting to note that when this item was subsequently included in the CFA analysis, it showed an acceptable standardised factor loading of 0.58 and 0.56 for the 3-factor and 2-factor models respectively. This implies that this item is clinically important for the complete assessment of CU traits in our population specifically under the *callousness* construct as *‘not taking the time to do things well’* could be contrived as having a callous attitude to other people especially those with higher authority, such as teachers or parents. Despite past studies on the Asian population mentioned above have opted for the short form version (12 items) of the ICU, with only the *callousness* and *uncaring* subscales (e.g., [24, 25]), it may not fully meet the purpose of why the ICU was created–which is to assess for DSM-5 specifier criterion (i.e., “concerned about performance at school, work, or in other important activities”, “shallow and deficient affect”) [53].

Further examination of the factors using CFA, with Model-1 being produced based on the three-factor model in EFA showed unsatisfactory fit analysis. Two items (Q2 and Q22) were subsequently deleted due to low factor loadings and this resulted in Model-2 which marginally improved the fit indices, but still did not reach a satisfactory level. Re-specifation of the model by adding correlated residuals for items 15 and 23, produced the final Model-3 and was deemed satisfactory with all fit indices achieving the acceptable threshold values. Such correlation between items had been employed in previous studies to achieve a better fit for the model indices [31]. While Hawes et al. concluded that the two items 15 and 23 displayed low discrimination parameters based on Item Response Theory (IRT) analysis and were subsequently removed from their final model [32]. Based on the number of modifications required to retain the items in the Malay-version ICU scale, it suggested that the relationship between the items in this translated version may be different than the original version.

In Model-4, the fit indices were not within the acceptable threshold values for CFI, TLI and WRMR. Two problematic items, Q2 and Q22 were removed from the model. The fit indices improved but still not within the acceptable threshold value. Model-5 was re-specified by adding meaningful correlation between two items’ residual (Q23 and Q15) and this resulted in Model-6. This re-specification method was supported by Kenny [54], where he suggested two strategies for re-specification to improve model fitting which both are best done a priori–first, by testing theoretically meaningful ways to complicate or to simplify the model, or second, by using modification indices and standardised residuals in re-specifying the model. The latter was used in this study. Model-6 fit the data well with all the fit indices achieving the recommended threshold value. According to Hair et al., for a scale with more than 12 items, a CFI and TLI of more than 0.92 is acceptable [45]. All items achieved satisfactory factor loading, except for item 5, which was then being removed. Most of the shortened versions of the ICU proposed by other studies have also excluded item 5 from their final model [31, 32, 51]. Based on the fit indices displayed in Model-3 and Model-6, this study has an acceptable fit to both the two-factor and three-factor models.

The final factors in the present study aligned almost similarly with those found by Roose et al. [22] and Essau et al. [30], only for callousness factor, item 22 load into *unemotional* subscale, while for *uncaring* factor, item 8 load into *callousness* factor. Pechorro et al. [23] analysis showed 9 items fitted into the *callousness* subscale, with the only difference from the present study was item 8 being in this factor rather than the *uncaring* factor. For the *unemotional* factor, items 1, 6, 14, 19, and 22 were retained in this factor.

The M-ICU final model is a valid and reliable tool to be used to assess adolescents’ CU traits which can be predictive of future behavioural issues and conduct disorder symptoms. Therefore, this would be beneficial to help screen and for early detection of any children or adolescents with elevated levels of CU traits, and to refer them for appropriate early interventions and psychological support [34]. It is noteworthy that heightened level of CU traits, which is often being underestimated, is usually a risk factor that can appear somewhat early in a person’s life and propel them down the road to be involved in a perpetual cycle of severe or chronic violence and antisocial behaviour. This could eventually lead to a huge burden on the resources of not just to the public health and criminal justice systems, but the economic and social welfare systems of the society at large [55]. The researchers thus would recommend the usage of the self-report M-ICU to measure the level of CU traits, especially among the vulnerable groups, in the hope of preventing potentially devastating consequences of antisocial behaviour. Additionally, the researchers would argue that this version of the M-ICU based on Model 6 (with two factors and 17 items) would be as effective in measuring CU traits as the original 24-item version, with an added advantage of being less time consuming.

### Limitations and recommendations

We acknowledge the findings in this study should be viewed in light of certain limitations. Firstly, there were no comparisons done with other tools measuring similar CU traits such as the Antisocial Process Screening device (APSD) or Youth Psychopathy Traits Inventory (YPI) to look at concurrent validity of the ICU-M [56]. This might add more value to the overall findings. It would also be beneficial to assess other significant measures commonly associated with pronounced CU traits such as levels of aggression using the Instrument for Reactive and Proactive Aggression (IRPA) self-report which has a validated Malay version [57] or even measures of Oppositional Defiant disorder (ODD) or Conduct disorder (CD) in children using scales such as the Diagnostic Interview Schedule for Children (DISC-IV) which has shown good reliability and validity [58].

The study was conducted via a cross-sectional design, which means other important psychometric properties such as test-retest reliability could not be tested. It would be recommended for future researchers to include a follow-up assessment in future research investigating the CU traits among Malaysian youth. It will also be useful to conduct a prospective study that will look at whether the extracted CU subfactors from this study remain stable in the same population during adolescence and when they enter early adulthood which would enhance the predictive validity of this scale. In addition, other internal and external validity tests should be conducted in future study to strengthen the quality of the translated scale of M-ICU.

In addition, only self-report version was tested in this study. Self-rated questionnaires could be prone to response bias when the respondents choose to rate themselves more positively. It might be prudent to measure the young people’s ICU by collecting data from other informants including parent and teacher to compare the influences of different informers, which may help to increase the accuracy of the overall ICU’s CFA validity. It is unclear whether the results and factor structure extracted from this study could be generalised to other samples in other states in Malaysia considering this study was conducted in the east coast city of Kuantan. However, there is generally good representation in terms of sex and ethnicity in the present study sample and the sample size was also adequate. It would be interesting to discover if the adolescent population in other states of Malaysia would display similar findings as there might be some variation in the Malay language capability of these adolescents. The two- and three-factor models of the M-ICU could also be utilised in studies regarding CU traits and correlations with other measures of aggression and antisocial behaviour in a more specialised population in Malaysia such as in the clinic settings, juvenile institutions or other referral-based settings.

## Conclusion

The present study, once again, supported that the M-ICU with two- or three-factor models as having good factor structure validity and satisfactory reliability, consistent with previous validation studies conducted on the ICU in different countries and languages. Nevertheless, it would be useful to replicate this study to further examine the item content of the M-ICU and also to see the replicability of this translated scale in different populations with more diverse age groups including younger children in primary school or even young adults (e.g., college and university students). Overall, the researchers would recommend the usage of the two-factor M-ICU scale for similar age group and among adolescents from the community sample based on Model-6 of the CFA, given that it has robust goodness of fit indices as compared to the three-factor ICU model. This was achieved by removing the unreliable items associated with the *unemotional* subscale. This is in tandem with multiple other studies especially in non-Western populations which have shown the low or non-reliability of the *unemotional* factor in measuring CU traits and the frequent negative correlations it displays with future conduct problems [14, 26, 29, 30]. This study has provided further evidence of the possibility of measuring CU traits with a modified ICU scale consisting of fewer and more precise items as compared to older versions of the ICU. To conclude, the findings show this scale can be useful in identifying the level of CU traits among school children. Screening these young people using the ICU can potentially help to identify those with heightened level of CU traits, who can then be given early and targeted intervention, which may prevent future conduct problems or antisocial behaviour.

## Supporting information

S1 Appendix (DOCX)

S1 Data (XLSX)
