# Supplementary material for: Validation of the inventory of Callous-Unemotional Traits among school-going adolescents in Malaysia
Source: PLoS One. 2023 Feb 16;18(2):e0276724. doi: 10.1371/journal.pone.0276724 (PMC9934360; doi:10.1371/journal.pone.0276724)
Supplement: S1 Appendix — (DOCX) [file pone.0276724.s001.docx]

# Appendix

**Inventory of Callous-Unemotional Traits – Youth Report (Malay Version)**

*Bagi setiap penyataan di bawah, bulatkan satu nombor yang paling sesuai untuk menggambarkan diri anda. Gunakan skala yang disediakan untuk menentukan nombor yang paling sesuai. Tandakan nombor untuk setiap pernyataan yang diberikan.*

| *Tidak benar sama sekali* | *Agak benar* | *Benar* | *Sangat Benar* |
| --- | --- | --- | --- |
| 0 | 1 | 2 | 3 |

| 1. | Saya menyatakan perasaan saya secara terbuka. |
| --- | --- |
| 2. | Apa yang saya anggap sebagai betul atau salah berlainan dari anggapan orang lain. |
| 3. | Saya mengambil berat tentang prestasi saya di sekolah atau tempat kerja. |
| 4. | Saya tidak peduli siapa yang saya sakiti untuk dapatkan apa yang saya mahu. |
| 5. | Saya rasa teruk atau bersalah apabila melakukan sesuatu yang salah. |
| 6. | Saya tidak menunjukkan emosi saya kepada orang lain. |
| 7. | Saya tidak peduli tentang menepati masa. |
| 8. | Saya prihatin tentang perasaan orang lain. |
| 9. | Saya tidak peduli jika saya mendapat masalah. |
| 10. | Saya tidak membiarkan perasaan saya mengawal diri saya. |
| 11. | Saya tidak peduli tentang melakukan sesuatu dengan baik. |
| 12. | Saya kelihatan tidak mesra dan tidak peduli tentang orang lain. |
| 13. | Saya mudah mengakui kesalahan diri sendiri. |
| 14. | Ia sangat mudah untuk orang lain mengetahui bagaimana perasaan saya. |
| 15. | Saya sentiasa mencuba yang terbaik. |
| 16. | Saya meminta maaf (“dengan berkata saya minta maaf”) kepada orang yang saya sakiti. |
| 17. | Saya cuba untuk tidak menyakiti perasaan orang lain. |
| 18. | Saya tidak rasa menyesal apabila saya melakukan sesuatu yang salah. |
| 19. | Saya sangat menyatakan perasaan secara terbuka dan beremosi. |
| 20. | Saya tidak suka meluangkan masa untuk melakukan sesuatu dengan baik. |
| 21. | Perasaan orang lain adalah tidak penting bagi saya. |
| 22. | Saya menyembunyikan perasaan saya daripada orang lain. |
| 23. | Saya bekerja keras dalam setiap perkara yang saya lakukan. |
| 24. | Saya melakukan sesuatu untuk membuat orang lain rasa gembira. |
